# Bioarcheology: Medicine, Biology, and Forensic Sciences

**DOI:** 10.1155/2015/671206

**Published:** 2015-08-06

**Authors:** Otto Appenzeller, Timothy G. Bromage, Rabab Khairat, Andreas G. Nerlich, Frank Jakobus Rühli

**Affiliations:** ^1^New Mexico Health Enhancement and Marathon Clinics Research Foundation, Albuquerque, NM 87122-1424, USA; ^2^Hard Tissue Research Unit, Department of Biomaterials & Biomimetics, New York University College of Dentistry, New York, NY 10010-4086, USA; ^3^Department of Medical Molecular Genetics, Division of Human Genome and Genome Research, National Research Center, Cairo, Egypt; ^4^Section of Paleopathology, Pathology Institute, Klinikum München-Bogenhausen, Munich, Germany; ^5^Institute of Evolutionary Medicine, University of Zurich, 8057 Zürich, Switzerland

*Bioarcheology* is the study of archived human and animal remains. The term was first proposed by the British archeologist Grahame Clark to denote the investigations of animal bones from archeological sites. Since its introduction in 1972, this term has been applied to encompass additional scientific domains and has also been used for the multidisciplinary papers collected in this special issue.

Agriculture's first appearance on the archeological horizon is a matter of debate. Evidence provided by multidisciplinary studies encompassing archaeobotany and zooarchaeology and the emergence of agricultural practices in the “fertile crescent” (a crescent shaped area containing fertile lands amidst semiarid regions of Western Asia, the Nile valley, and northeast Africa) in Iran add to the science of ancient agriculture (S. Riehl et al.).

Egyptian archeology has been a widely accepted aspect of bioarcheology. Here a wealth of information has been collected with the applications of new techniques to ancient tissues such as X-ray analyses applied in the field to archived human bones (F. Rühli et al.).

Mandibles excavated from Qubbet el-Hawa cemetery in Aswan, Egypt, have yielded information on the food consumed by middle class people during several periods of ancient Egyptian history. The elemental analysis of the bones hinted at social and climatic changes affecting ancient populations and shows the effects of state socialism (a variant socialism in which the power of the state is employed to creating an egalitarian society through the control of production of all goods) and agricultural conditions during the 17th Dynasty in Upper Egypt (G. D. AL-Khafif and R. El-Banna).

South American frozen mummies from mountain tops of the high Andes have only recently become available for bioarcheological studies. Such studies have revealed important aspects of human sacrificial practices in pre-Hispanic times. Because of the exceptional preservation of the soft tissues of these mummies due to the intense cold and ambient dryness at altitudes at which they were found, important human pathologies accompanying life under Inca rule have become accessible to modern techniques (M. C. Ceruti).

The histology of soft tissues is a well-accepted diagnostic method in modern human pathology. However, its application to ancient tissues brings challenges to the standard interpretation of histological stains because of the variable preservation of specimens depending on numerous unforeseeable factors. The postmortem interval before histological study is one crucial obstacle. The longer this time the less dependable the results. Paleopathological examinations provided guidelines to the tissues likely to be best preserved and allowed the most reliable diagnoses (C. Grove et al.).

Successful and cheap methods recently developed for the extraction of ancient DNA (aDNA) have led to important studies in evolution and worldwide human migrations. East Asian mitochondrial DNA (mtDNA) especially of Koreans has been used to determine single nucleotide polymorphisms in mtDNA in human bone samples from premodern Joseon tombs in Republic of Korea (C. S. Oh et al.).

Archeological materials useful for the study of bioarcheology are conserved in museums throughout the world. Curators prefer the study of their collections by noninvasive methods. The desire to maintain specimens intact is shared by bioarcheologists too. Nevertheless, invasive methods such as autopsies, endoscopies, and biopsies are sometimes unavoidable for accurate diagnosis. In this setting controversies are unavoidable but scientific criteria coupled with ethical considerations are proposed which may resolve disputes (D. Moissidou et al.).

Monastic inhabitants of ancient institutions provide fertile grounds for the investigations of diseases and social aspects of a particularly privileged population. Regional differences are not often revealed but when such studies are restricted to defined populations such as those in Bavaria, Germany, insights into paleopathology, nutrition, and lifestyles of a well-defined human group become apparent (A. G. Nerlich et al.).

Statistical modeling has advanced science in general and bioarcheology in particular. Growth lines in archived biological materials such as teeth and hair proved valuable in providing evidence of metabolic health, physiology, and lifestyles of long dead individuals. Thus new noninvasive methods for the analyses of curated archeological specimens could avoid ethical consideration in bioarcheological investigations (C. Qualls and O. Appenzeller).

Computational methods could also predict the physical appearance, mood, and clinical diagnosis such as hirsutism (excessive hair growth) also known in some individuals as Cantú syndrome (C. Qualls et al.).

Recent massive sequencing of ancient DNA has transformed the study of human evolution and human history. Such studies are only now beginning in bioarcheology but important insights into modern diseases are likely to be gleaned from analyses of bioarcheological specimens.

